# The whole transcriptome analysis using FFPE and fresh tissue samples identifies the molecular fingerprint of osteosarcoma

**DOI:** 10.3389/ebm.2024.10161

**Published:** 2024-06-20

**Authors:** Bal Hari Poudel, Sulev Koks

**Affiliations:** ^1^ Center for Molecular Medicine and Innovative Therapy, Murdoch University, Perth, WA, Australia; ^2^ Perron Institute of Neurological Diseases, Perth, WA, Australia; ^3^ Central Department of Biotechnology, Tribhuvan University, Kathmandu, Nepal

**Keywords:** osteosarcoma, osteogenic sarcoma, transcriptome, RNA sequencing, differential gene expression

## Abstract

Osteosarcoma is a form of bone cancer that predominantly impacts osteoblasts, the cells responsible for creating fresh bone tissue. Typical indications include bone pain, inflammation, sensitivity, mobility constraints, and fractures. Utilising imaging techniques such as X-rays, MRI scans, and CT scans can provide insights into the size and location of the tumour. Additionally, a biopsy is employed to confirm the diagnosis. Analysing genes with distinct expression patterns unique to osteosarcoma can be valuable for early detection and the development of effective treatment approaches. In this research, we comprehensively examined the entire transcriptome and pinpointed genes with altered expression profiles specific to osteosarcoma. The study mainly aimed to identify the molecular fingerprint of osteosarcoma. In this study, we processed 90 FFPE samples from PathWest with an almost equal number of osteosarcoma and healthy tissues. RNA was extracted from Paraffin-embedded tissue; RNA was sequenced, the sequencing data was analysed, and gene expression was compared to the healthy samples of the same patients. Differentially expressed genes in osteosarcoma-derived samples were identified, and the functions of those genes were explored. This result was combined with our previous studies based on FFPE and fresh samples to perform a meta-analysis. We identified 1,500 identical differentially expressed genes in PathWest osteosarcoma samples compared to normal tissue samples of the same patients. Meta-analysis with combined fresh tissue samples identified 530 differentially expressed genes. *IFITM5, MMP13, PANX3*, and *MAGEA6* were some of the most overexpressed genes in osteosarcoma samples, while *SLC4A1, HBA1, HBB, AQP7* genes were some of the top downregulated genes. Through the meta-analysis, 530 differentially expressed genes were identified to be identical among FFPE (105 FFPE samples) and 36 fresh bone samples. Deconvolution analysis with single-cell RNAseq data confirmed the presence of specific cell clusters in FFPE samples. We propose these 530 DEGs as a molecular fingerprint of osteosarcoma.

## Impact statement

Although rare, osteosarcoma attracts global attention because of the unsatisfactory outcomes associated with current treatment approaches. In our investigation, we examined total RNA extracted from 90 FFPE paired samples, consisting of 50 tumoral bone specimens and their matched non-tumoral counterparts, sourced from osteosarcoma patients. By comparing our findings with previous studies, we uncovered differences in gene expression patterns between normal and affected bone tissues, particularly emphasizing changes in the regulation of collagen and extracellular matrix degradation and cell cycle regulation. These findings deepen our understanding of osteosarcoma and provide potential directions for future research endeavors.

## Introduction

Bone cancer (osteosarcoma, OS) is the most common primary tumour of the bone in children and young adults [1, 2]. Osteosarcoma arises from the cells forming bone tissue and can be found in the bone’s metaphysis, the region where the growth plate is located. The exact cause of osteosarcoma is not fully understood, but several risk factors have been identified [3]. Some individuals may have a genetic predisposition to developing osteosarcoma, although it is not typically inherited in a Mendelian pattern. Certain preexisting conditions, such as hereditary retinoblastoma (a rare eye cancer) and Paget’s disease of bone, have been linked to an increased risk of osteosarcoma [4].

Symptoms of osteosarcoma may include pain and swelling in the affected bone or joint, limited range of motion in the nearby joint, a mass or lump in the affected area, fractures or bone weakening in the affected bone [5, 6]. Diagnosis typically involves a combination of imaging studies like X-rays, MRI, and CT scans, as well as a biopsy to confirm the presence of cancerous cells [5, 6]. Treatment for osteosarcoma usually involves a multimodal approach, which may include surgery, chemotherapy and radiation therapy [6], however, none of them are accurate and also have strong adverse effect with chemotherapy and radiation therapy. In addition, there is higher chances of getting a second cancer [7, 8].

The prognosis for osteosarcoma varies depending on factors such as the extent of the disease, response to treatment, and the presence of metastasis [9]. Early diagnosis and aggressive treatment can significantly improve the chances of successful outcomes. Long-term follow-up care is essential to monitor for any potential recurrence or late effects of treatment. Treatment plans are usually developed in collaboration with a team of oncologists, surgeons, and other healthcare professionals.

Whole transcriptome analysis is a powerful tool used in modern-day molecular biology to study the entries set of RNA molecules produced by a cell or a population of cells [10, 11]. Transcriptome analysis provides snapshots of the genes expressed and can be compared with normal [11]. However, the differentially expressed genes (DEGs) may vary from sample to sample according to the quality of the samples. For rare diseases like osteosarcoma, there are not many options for getting fresh samples due to the death of patients and getting fresh bone samples is not easy; hence, formalin-fixed paraffin-embedded tissues (FFPEs) are the best way to analyse the sample. However, RNA gets degraded [12] due to formalin fixation in FFPE.

In this paper, we report a complex transcriptomic analysis that is based on four independent studies. We performed two original experiments and combined these results with previously published studies. This study provides the molecular signature of osteosarcoma to generate fundamental knowledge to develop new drugs against this disease. Through this study, we compared the DEGs in FFPE with fresh bone samples to identify whether or not the FFPE DEGs aligned with the fresh bone samples. We performed RNAseq analysis on a new set of 90 samples. We later compared it to the two other independent studies (FFPE and fresh) to conduct a meta-analysis of OS transcriptome. Identified molecular mechanisms and genes could be used as a potential target for osteosarcoma drug development and will be evaluated in future studies.

## Materials and methods

### The sample processing and whole transcriptome analysis

Our original sample analysis is based on two independent analyses of the formalin-fixed paraffin-embedded (FFPE) samples. The paraffin blocks were cut, and RNA was extracted. This way, any archived biological samples can be analysed for rare diseases like osteosarcoma; using FFPE samples is the only option to get meaningful and large samples for the complex transcriptomic analysis. The FFPE samples (N = 90: 50 OS + 40 healthy control) were obtained through PathWest Nedlands (QEII Medical Centre, Australia) and were processed in two batches, and total RNA was extracted using the Norgen FFPE RNA purification kit (#25300) using the manufacturer’s standard protocol. The purified RNA samples were sent to the Australian Genome Research Facility (AGRF) Melbourne for sequencing also in two separate batches.

### FFPE sample analysis

We combined two PathWest FFPE studies (90 samples altogether) into a single analysis to increase the formal statistical power of the analysis. Details of the samples are shown in Supplementary Table S1. Briefly, this table highlights the gender, sample group (tumour or normal), age at onset, deceased or alive and chemotherapy or non-chemotherapy. The raw FASTQ files obtained after sequencing were used for the data analysis. DEGs were detected by comparing the OS with normal samples using Salmon [13]. Statistical analysis of data and differential gene expression was also performed by using the DESeq2 package of R [14, 15]. The magnitude of the differential gene expression between tumour and healthy samples was calculated by analysing the log-2-fold change of the genes (logFC, the cut-off value of 0.5). Benjamini-Hochberg method available in the DESeq2 package was used to adjust the nominal *p*-value (padj) in order to correct against false positive findings caused by multiple tests. The significance level was set at padj<0.05 [15].

The results from this study were used in the further meta-analysis and combined with two previously published data.

### Meta-analysis

The results from four different whole transcriptome studies of osteosarcoma were combined to identify the differentially expressed genes. Two studies are from the present analysis, two others are from our previously published papers. Three studies were based on FFPE samples and one previous study from Estonia and Vietnam was based on fresh OS samples [16]. (Study 1: Estonia + Vietnam FFPE samples N = 15, Study 2: PathWest FFPE 1: N = 24 OS + 16 healthy controls, Study 3: PathWest FFPE 2N = 26 OS + 24 healthy controls and Study 4: Estonia and Vietnam fresh bones N = 18 OS + 18 healthy controls). The DEGs found in individual studies were compared to each other to find common DEGs.

### Deconvolution with single-cell transcriptomics

Deconvolution was based on a previously published single-cell transcriptomic study in OS [17]. Cell profiles in scRNAseq study were from six OS patient samples (GSE162454). ‘Seurat’ package was used to find conserved markers that define cell clusters. To identify canonical cell type marker genes that are conserved across all conditions (tumour and treatment), we used the “FindConservedMarkers ()” function. This function tests differential gene expression for each dataset/group and combines the *p*-values using meta-analysis methods from the “MetaDE” R package [18]. “Granulator” package was used to identify the cluster-specific profiles in bulk FFPE RNAseq data.

### Functional analysis of the differentially expressed genes

To understand the variation of different groups of gene functions in osteosarcoma, gene ontology (GO) analysis and Kyoto Encyclopedia of Genes and Genomes (KEGG) were used to interpret the change in gene expression along with their cellular locations, biological processes, and involvement in the molecular pathway [19, 20].

## Results

### FFPE RNA-seq and meta-analysis

**FIGURE 1:**
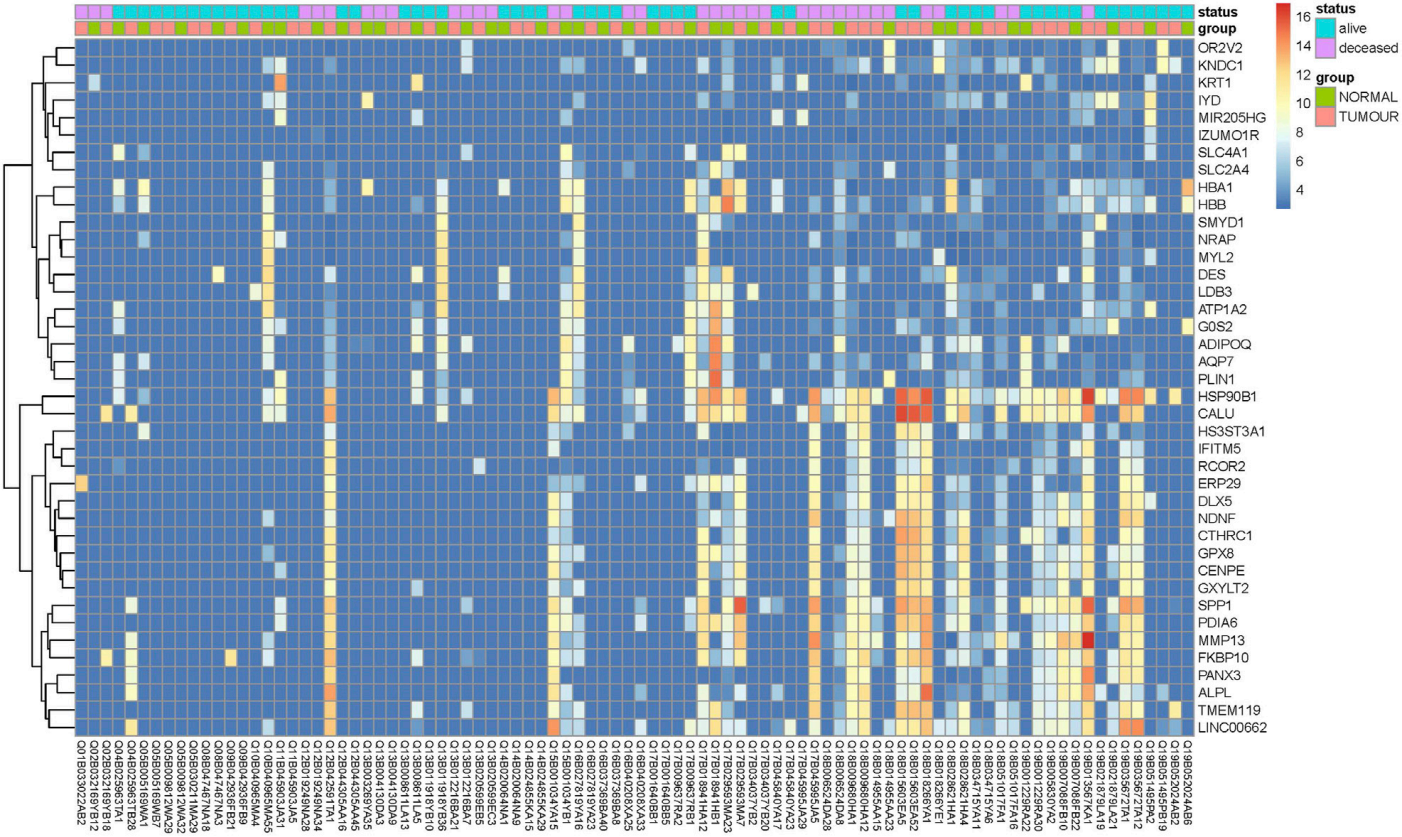
Heat map of top differentially expressed genes with largest fold change differences between tumor and normal samples.

**FIGURE 2:**
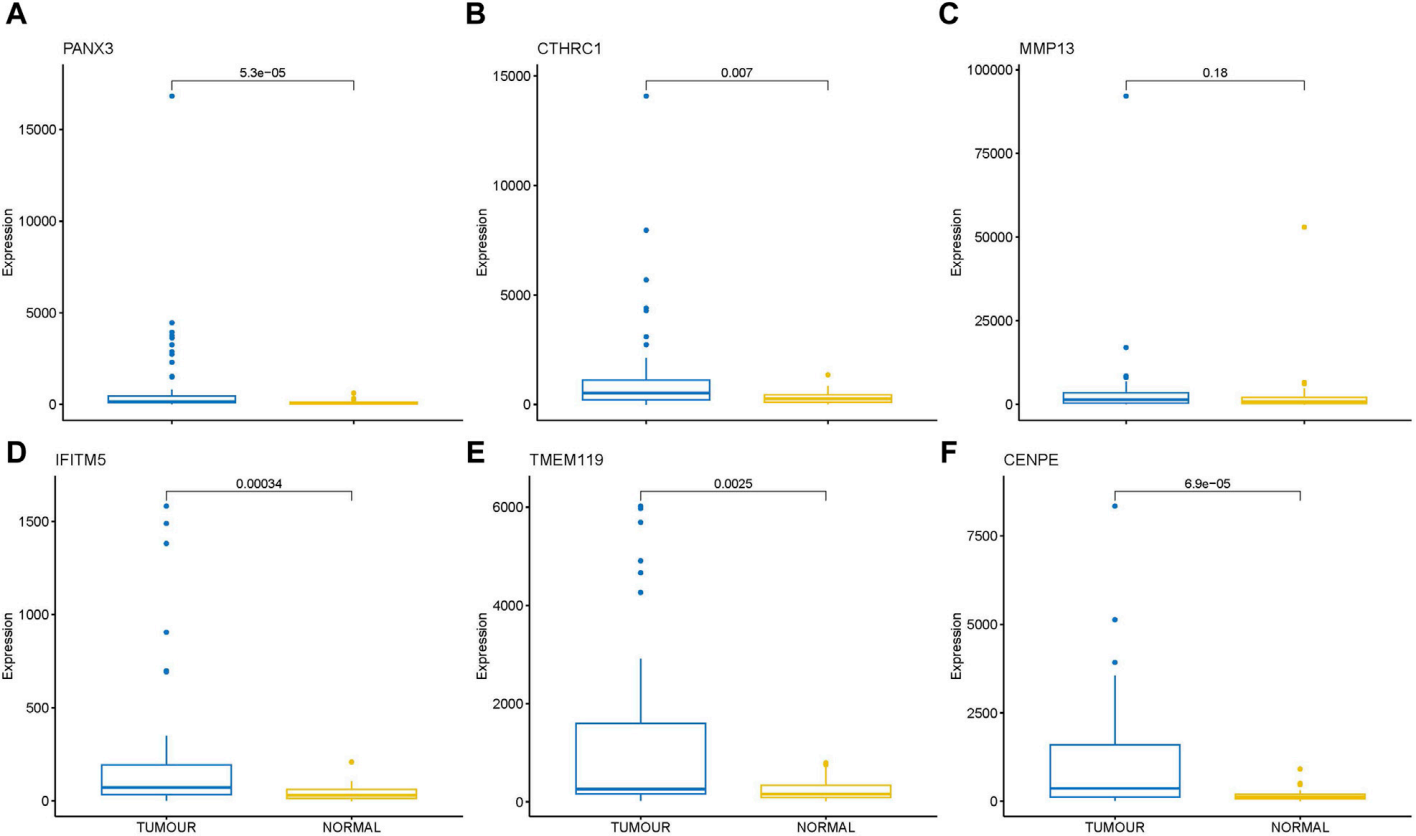
Box plot showing direct comparison of gene expression in tumor and normal samples for some upregulated genes, gene expressions are presented in normalized count. **(A)** PANX3 **(B)** CTHRC1 **(C)** MMP13 **(D)** IFITM5 **(E)** TMEM119 **(F)** CENPE.

**FIGURE 3:**
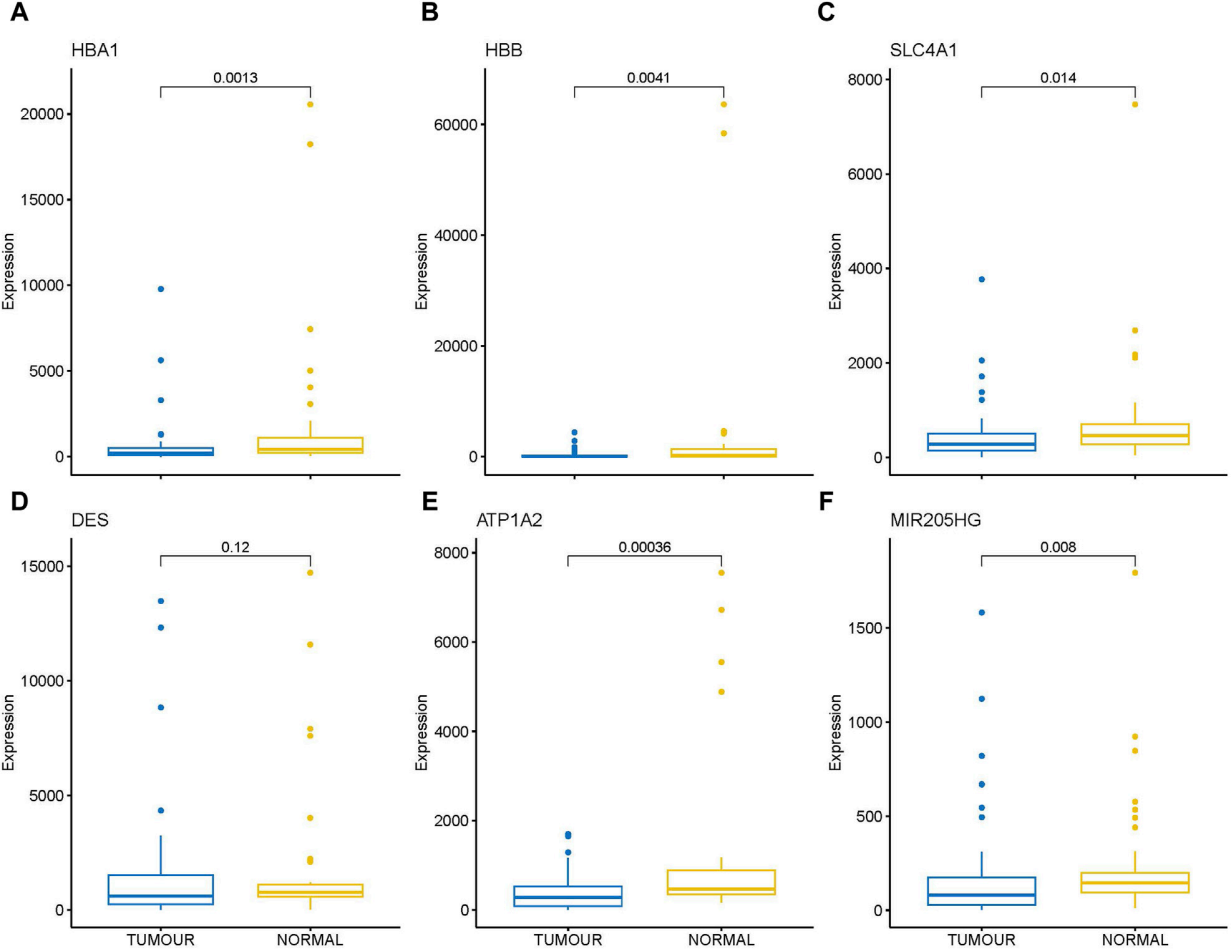
Box plot showing direct comparison of gene expression in tumor and normal for some down regulated genes, gene expressions are presented in normalized count. **(A)** HBA1 **(B)** HBB **(C)** SLC4A1 **(D)** DES **(E)** ATP1A2 **(F)** MIR205HG.

Transcriptome analysis of the PathWest FFPE samples showed a large number of differentially expressed genes in tumours compared to healthy bone, as shown in Table 1 and in Figure 1 (heatmap of the top genes form the FFPE 90 sample). Some of the overexpressed genes were *MMP9, CCN4, HAPLN1, ALPL, MMP13, PANX3, IFITM5, , CTHRC1, , TMEM119,*. In addition, *COLLA11A1, FBN2, IBSP, COLA10A1, MDFI, GPX8* were also found to be overexpressed. A comparison of gene expressions in normal and tumours for some of the top differentially (upregulated and downregulated) expressed genes is presented in Figures 2, 3.

**TABLE 1:** List of top highly upregulated genes compared to normal tissue.

Gene name	Symbol	Base mean	log2FoldChange	Padj
Matrix metallopeptidase 9	MMP9	707.4	7.5	4.12E-25
Cellular communication network factor 4	CCN4	238.0	6.8	5.30E-24
Hyaluronan and proteoglycan link protein 1	HAPLN1	70.6	6.4	1.12E-24
Alkaline phosphatase, biomineralization associated	ALPL	158.1	6.4	8.19E-23
Matrix metallopeptidase 13	MMP13	276.9	6.3	8.61E-21
Pannexin 3	PANX3	117.7	6.1	9.43E-21
Rhomboid like 2	RHBDL2	51.6	6.0	8.19E-23
Collagen triple helix repeat containing 1	CTHRC1	123.4	6.0	8.92E-20
Glucosamine-phosphate N-acetyltransferase 1	GNPNAT1	137.6	5.9	1.41E-19
Transmembrane O-mannosyltransferase targeting cadherins 2	TMTC2	134.7	5.9	1.41E-19
Anillin, actin binding protein	ANLN	91.7	5.9	3.21E-21
Collagen type XXIV alpha 1 chain	COL24A1	61.4	5.8	3.90E-21
Collagen type XI alpha 1 chain	COL11A1	824.5	5.8	3.52E-14
Interferon induced transmembrane protein 5	IFITM5	32.5	5.7	1.04E-21
DLG associated protein 5	DLGAP5	41.9	5.7	9.00E-23
Integrin binding sialoprotein	IBSP	263.7	5.7	1.63E-16
Parathyroid hormone 1 receptor	PTH1R	100.8	5.7	2.45E-18
Lymphoid enhancer binding factor 1	LEF1	75.1	5.6	3.83E-20
Proline rich 11	PRR11	115.6	5.6	3.84E-18
SRY-box transcription factor 11	SOX11	53.2	5.5	8.81E-21
Cyclin B2	CCNB2	31.6	5.5	2.03E-22
DNA polymerase eta	POLH	99.4	5.5	1.12E-19
Fibrillin 2	FBN2	159.2	5.4	1.04E-15
Sp7 transcription factor	SP7	169.0	5.4	9.98E-16
Spindle apparatus coiled-coil protein 1	SPDL1	62.2	5.4	1.28E-19
Sterile alpha motif domain containing 5	SAMD5	182.1	5.3	1.16E-14
Collagen type X alpha 1 chain	COL10A1	103.3	5.3	6.69E-16
MyoD family inhibitor	MDFI	72.7	5.3	2.02E-17
Kinetochore scaffold 1	KNL1	149.9	5.3	4.68E-16
Neuron derived neurotrophic factor	NDNF	208.4	5.2	5.31E-14

**FIGURE 4:**
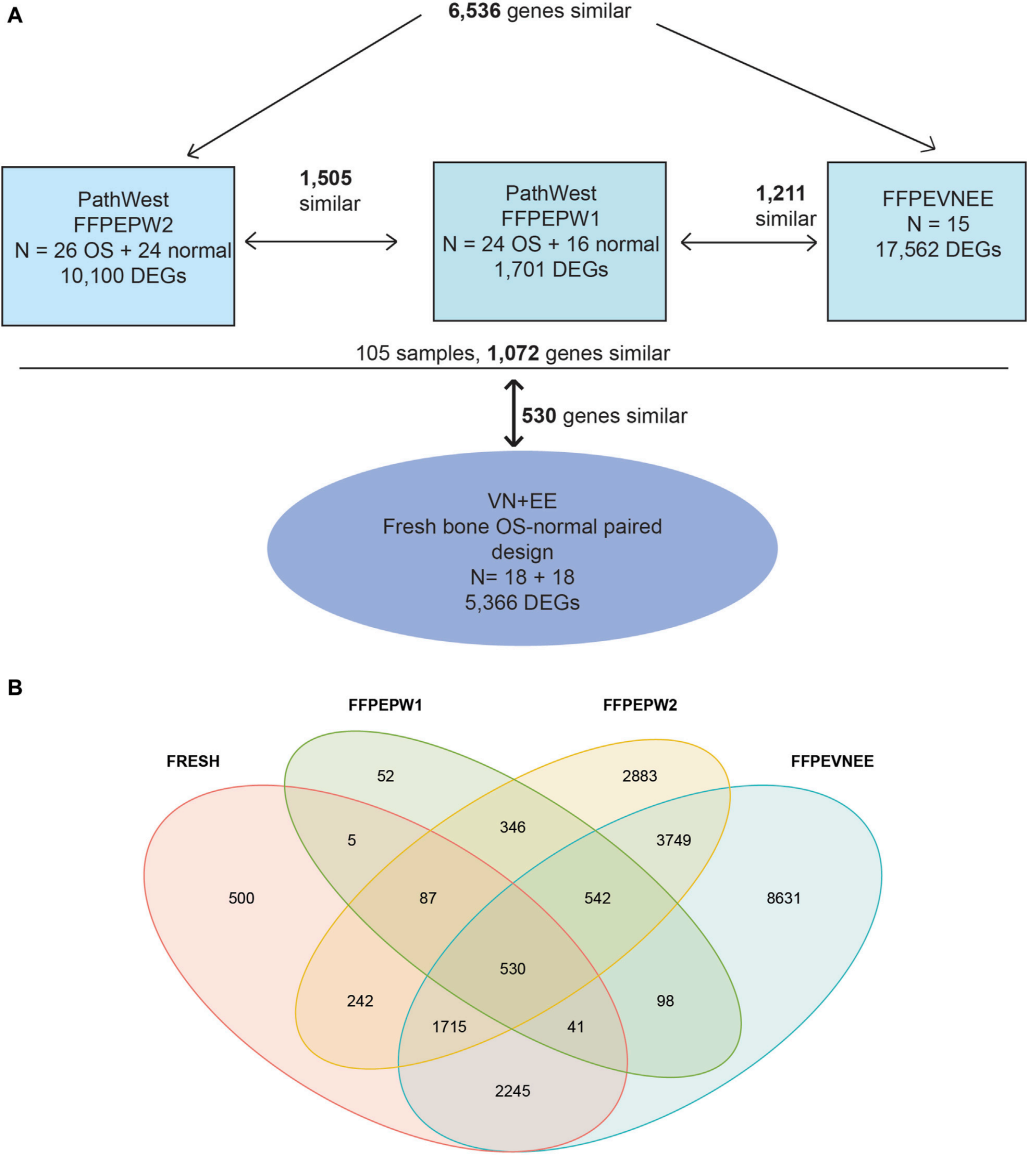
The meta-analysis. **(A)** Meta analysis design. Three formalin fixed paraffin embedded tissue-based study (FFPEVNEE, FFPEPW1, FFPEPW2) and one study with fresh bone samples were combined to identify the differentially expressed genes in osteosarcoma. VNEE–samples from Estonia and Vietnam B **(B)** Venn diagram showing the similarity and uniqueness of differentially expressed genes across four different studies.

For the meta-analysis, two PathWest studies were treated as independent studies because the samples were collected, extracted and sequenced independently from each other. During the meta-analysis we observed 6,536 DEGs similar among our PathWest FFPE 2 study and EE-VN study. However, the number of overlapping DEGs was smaller (1,505) in PathWest FFPE 2 and PathWest FFPE 1 (Figures 4A, B). In addition, 1,211 DEGs were similar among PathWest FFPE1 and EE-VN FFPE study. Among those 105 FFPE samples from three studies, we observed 1,072 identical DEGs. The DEGs observed with fresh bone OS samples were then compared with combined FFPE samples, and we observed 530 similar genes to be differentially expressed as indicated in Figure 4, and the number of similar and unique genes across different samples are presented in the Venn diagram (Figure 4B). Some of the top differentially expressed genes observed in OS compared to normal samples are presented in Figure 2. The clustering shows that OS has a remarkable transcriptome heterogeneity and indicates the molecular heterogeneity in the pathogenetic mechanisms causing the osteosarcoma. Our previous study found similar heterogeneity with only fresh tissue OS samples. Malignancies are known to have multiple mutations and complex molecular mechanisms that drive pathogenesis.

**FIGURE 5:**
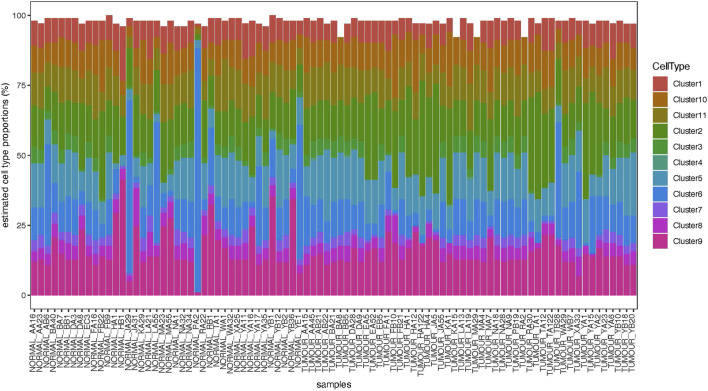
Proportions of cell clusters in different normal and tumour samples.

**FIGURE 6:**
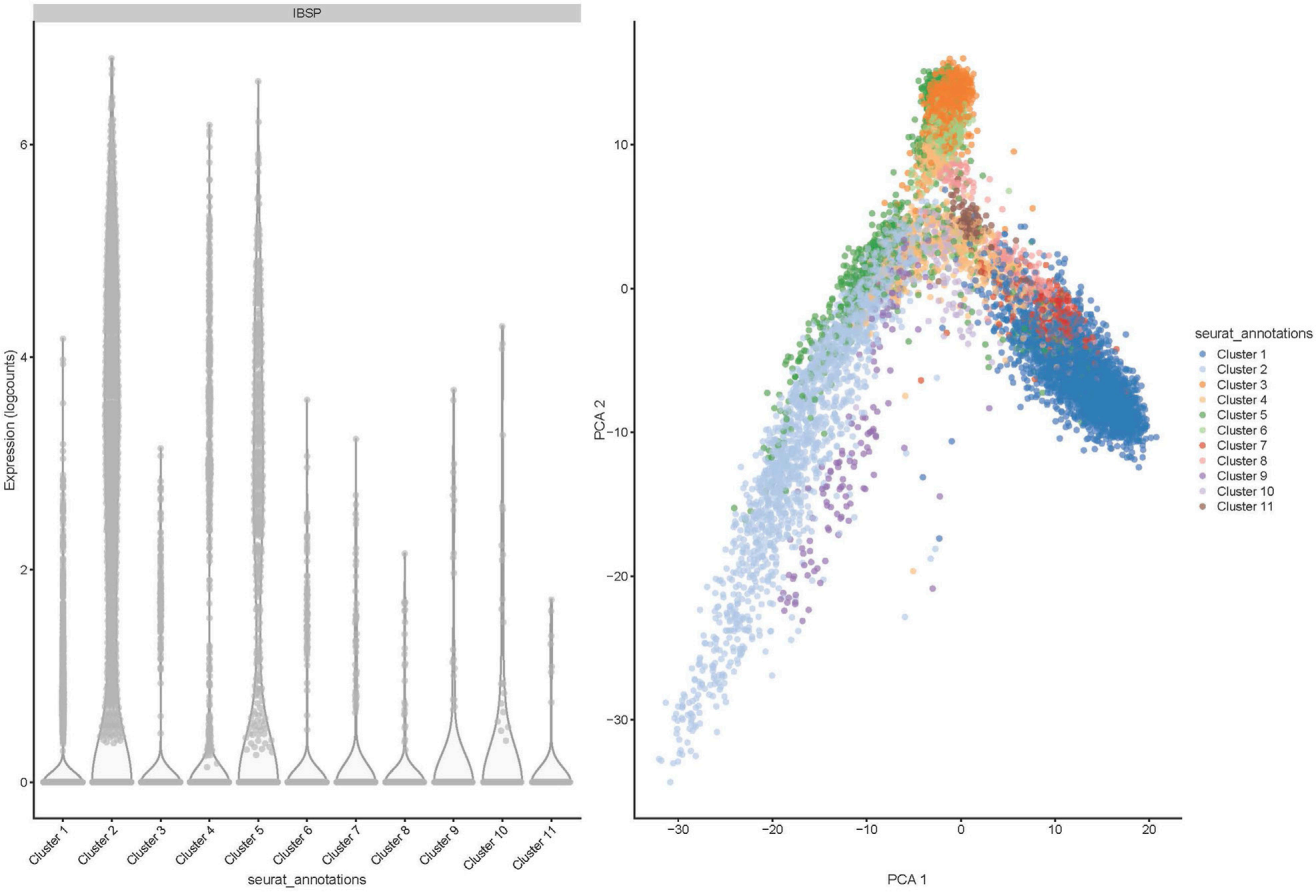
Distribution of 11 clusters of cells (shown in PCA1) and bone sialoprotein or integrin-binding sialoprotein (IBSP) expression in 11 different cell clusters shown as logcounts.

Deconvolution analysis was performed to identify the cellular populations from the FFPE-derived bulk RNAseq. Using the Seurat package and publicly available scRNA seq data from the OS sample, we could identify 11 clusters characteristic of the OS (Figures 5, 6). The clusters had specific patterns that could be designated to different cell populations, and cluster 2 was confirmed to be osteoblast-specific as it had typical osteoblast markers expressed. Our 90 samples from the Pathwest FFPE collection all had a stable proportion (20%) of osteoblastic cells Figure 5. Cluster 9 varied quite a lot between samples. The exact identity of these cellular clusters needs further analysis, but remarkably, deconvolution is possible from the FFPE-based bulk RNAseq data.

**FIGURE 7:**
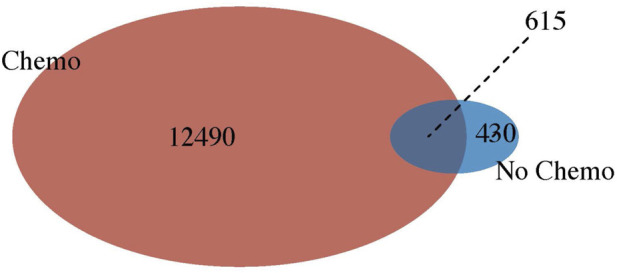
Venn diagram showing relationship between chemo-therapy verses non chemo-therapy.

In addition, we compared the differential gene expression among the PathWest FFPE samples for chemo and non-chemo and observed 615 common DEGs (Figure 7). These genes are not affected by chemotherapy and are specific for the pathogenesis of the OS. Chemotherapy itself affected a large number of genes and could be a significant confounding factor. This is why meta-analysis is needed to combine the statistical outcomes from independent studies. It is almost impossible to get clinical samples without treatment effects, and this is a common challenge for all real-life data-based studies, including genomics. We also observed many genes to be downregulated in OS compared to normal, as shown in Table 2. Some of the downregulated genes were SLC4A1, HBA1, HBA2, HBB, DES, ATP1A2, KRT1, LDB3, AQP7, FosB, G0S2, NRAP, and PLIN4, as shown in Table 2 with a log2FC value.

**TABLE 2:** Top downregulated genes in osteosarcoma based on FFPE PathWest 2 study.

Gene name	Symbol	Base mean	Log2FoldChange	Padj
Hemoglobin subunit alpha 1	HBA1	133.6	−6.3	7.21E-25
Regulator of G protein signaling 6	RGS6	13.9	−4.2	2.36E-17
ATPase Na+/K+ transporting subunit alpha 2	ATP1A2	44.0	−4.1	2.62E-11
Keratin 2	KRT2	10.7	−4.0	1.51E-13
Hemoglobin subunit beta	HBB	61.0	−4.0	3.33E-11
LIM domain binding 3	LDB3	40.4	−3.9	8.24E-11
Desmocollin 1	DSC1	9.8	−3.9	5.88E-14
Polymeric immunoglobulin receptor	PIGR	9.6	−3.8	2.33E-13
Myosin binding protein C1	MYBPC1	29.4	−3.7	8.11E-09
Keratin 1	KRT1	7.7	−3.6	6.32E-13
WNK lysine deficient protein kinase 2	WNK2	23.3	−3.3	5.73E-09
Perilipin 4	PLIN4	19.8	−3.3	1.46E-09
Adenylate cyclase 5	ADCY5	14.2	−3.3	1.06E-09
GPR176 divergent transcript	GPR176-DT	10.1	−3.3	5.11E-11
Hemoglobin subunit alpha 2	HBA2	40.6	−3.2	1.08E-07
XK related 4	XKR4	20.8	−3.2	1.36E-08
Complement C7	C7	20.2	−3.1	1.22E-07
Cardiomyopathy associated 5	CMYA5	65.3	−3.1	1.12E-06
Fatty acid binding protein 4	FABP4	10.0	−3.1	4.29E-10
FosB proto-oncogene, AP-1 transcription factor subunit	FOSB	227.3	−3.1	1.55E-05
Tubulointerstitial nephritis antigen like 1	TINAGL1	27.2	−3.1	1.27E-07
G0/G1 switch 2	G0S2	19.4	−3.0	1.86E-08
Adiponectin, C1Q and collagen domain containing	ADIPOQ	36.3	−3.0	1.31E-06
Synaptophysin like 2	SYPL2	12.0	−3.0	2.06E-08
Potassium voltage-gated channel subfamily A regulatory beta subunit 1	KCNAB1	14.8	−3.0	3.75E-08
Hemicentin 2	HMCN2	17.9	−2.9	1.87E-07
G protein-coupled receptor class C group 5 member A	GPRC5A	19.4	−2.8	3.85E-07
Ceruloplasmin	CP	12.2	−2.8	1.30E-07
Enamelin	ENAM	8.7	−2.8	3.91E-09
Sorbin and SH3 domain containing 1	SORBS1	60.6	−2.8	6.77E-06

**FIGURE 8:**
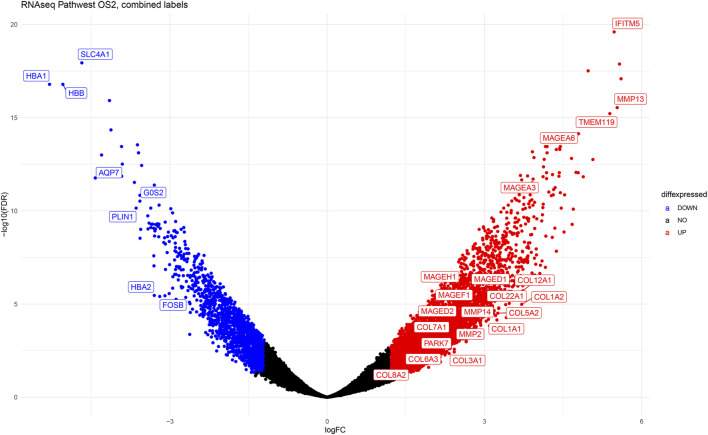
The volcano plot illustrating the differentially expressed genes in osteosarcoma. The y-axis represents -Log10 (FDR) values and x-axis represents Log2-fold -change (Log2Fc). Each spot represents a gene on the graph. The blue dots represent downregulated genes and the red dots on the right are upregulated genes.

The volcano plot (Figure 8) shows the top highly expressed and top downregulated genes in osteosarcoma compared to normal samples, with some of the genes labelled and indicated on the plot. This gives a good overview of the extent and statistical significance of the differentially regulated genes in all studies combined and illustrates the transcriptomic fingerprint of osteosarcoma.

### Functional analysis

**FIGURE 9:**
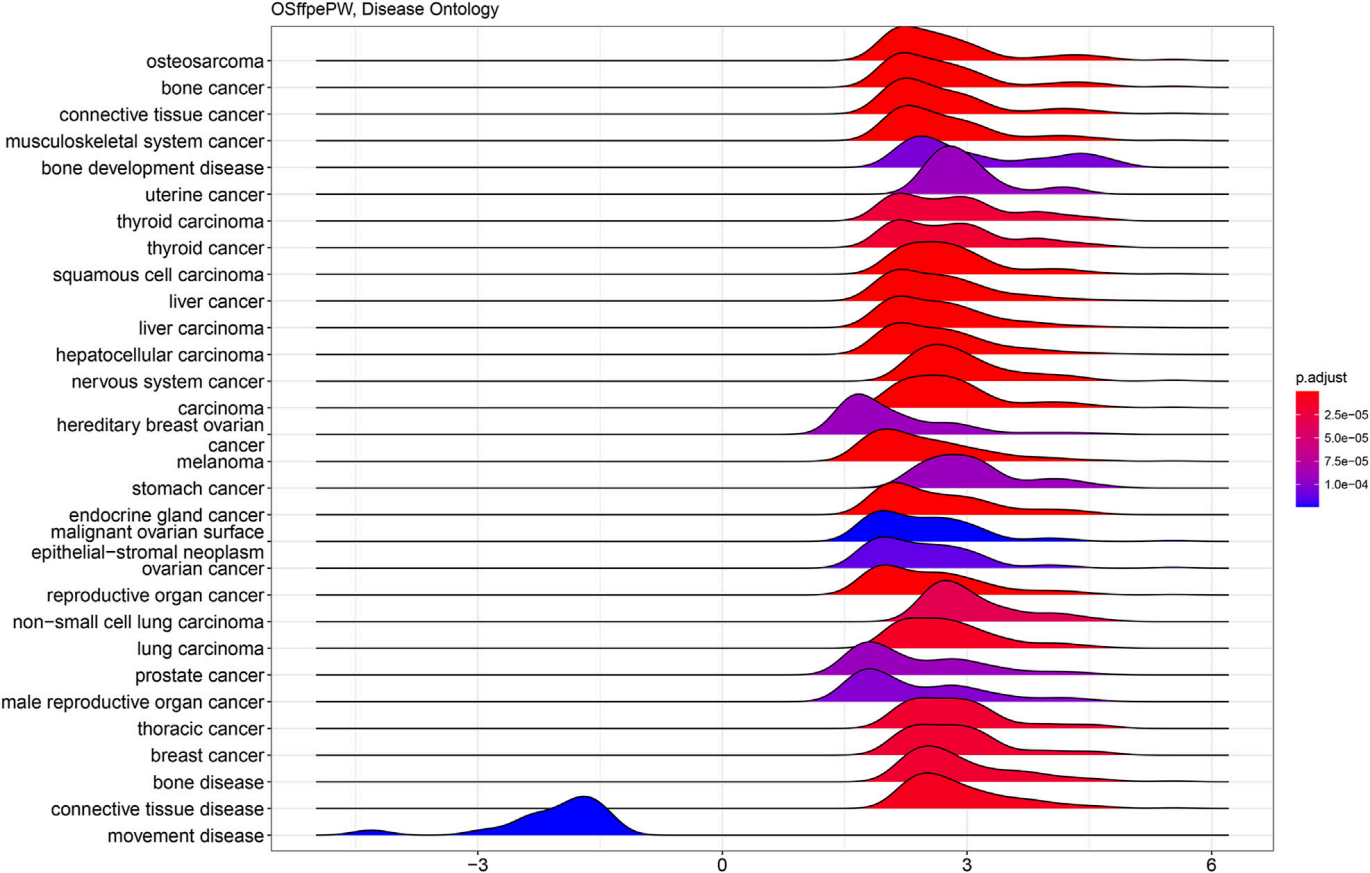
Ridge plot showing disease ontology analysis of PathWest samples. The y-axis represents -Log10 (FDR) values and x-axis represents *p*-value (p adjust). The blue ridge shown in left is correlated with downregulated genes and the ridges on right observed for upregulated genes. Related diseases are shown through X-axis.

GO and KEGG analyses were carried out to investigate the activation of the cellular pathways and common functional themes activated by the differentially expressed genes. This analysis is based on the differences among gene groups with various expressions and provides a preliminary interpretation of the biological activities of the differentially expressed genes. Similarly, disease ontology was performed to identify the disease patterns in the differentially expressed genes. We identified that the gene expression pattern we found matches with the osteosarcoma verifying our results again. A disease ontology study showed (Figure 9) that the DEGs are highly associated with osteosarcoma, bone cancer, connective tissue cancer, bone development disease and many other cancers (Figure 9) and also observed to be correlated with movement disease.

Functional analysis was done to identify the cellular and molecular pathways that had changed in the OS samples. This lets us identify the common themes that connect these genes in this list.

**FIGURE 10:**
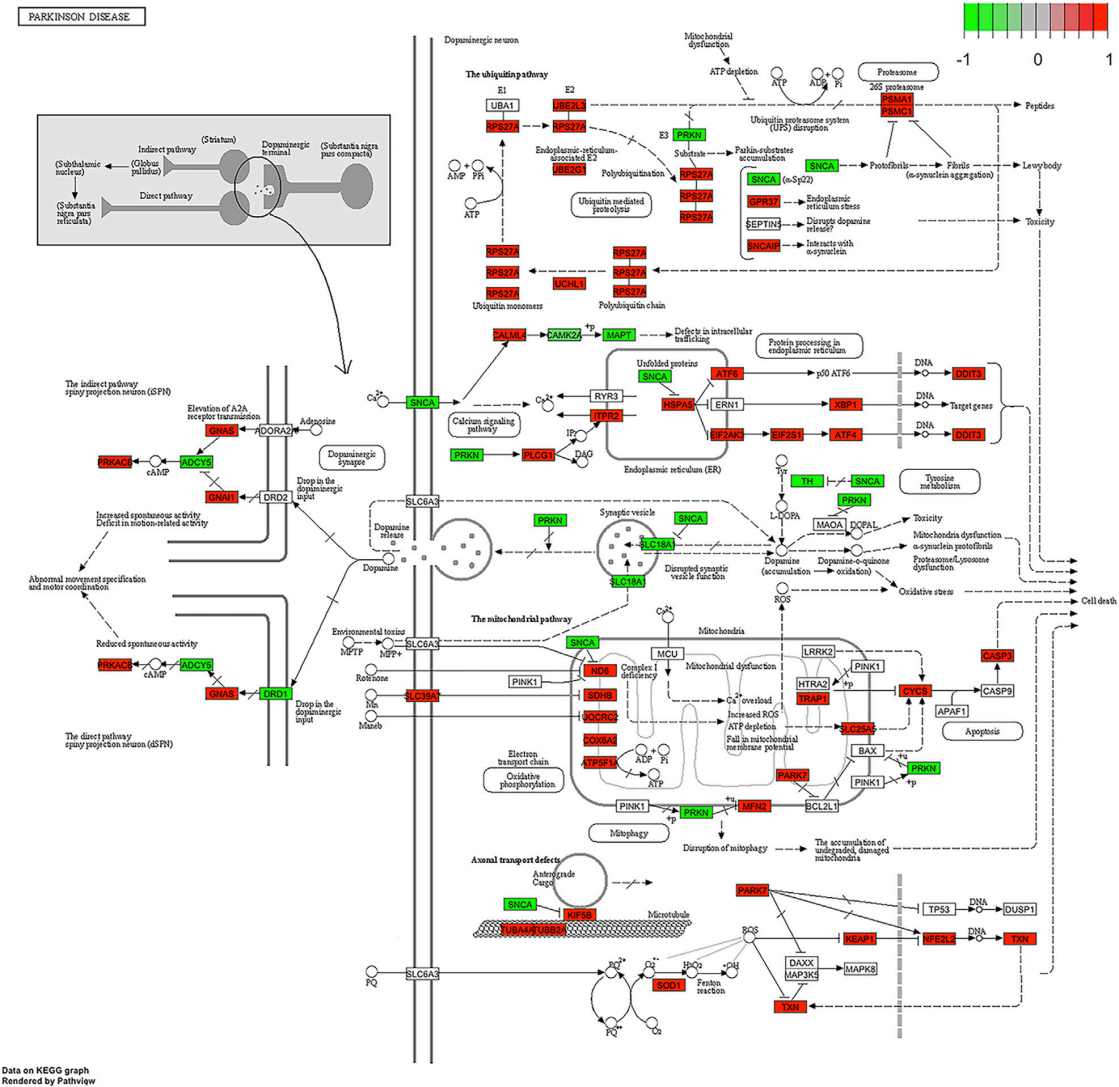
Parkinson disease pathway. The roles of PARK7 protein in Parkinson disease are highlighted.

KEGG pathway analysis is shown in Figure 10, and it describes the activation of the ribosome and protein processing in the endoplasmic reticulum. The KEGG pathway system is a unified canonical system to develop pathway maps for different cellular and biological functions. These maps comprehensively describe variable biological functions and provide maps for visualisation. The maps help us to define these molecular functions that are affected by osteosarcoma. KEGG pathway analysis also identified viral carcinogenesis pathways with some immune-regulated pathways.

Interestingly, a Parkinson’s disease-related pathway was also identified (Figure 10). This finding possibly reflects the overlap between protein processing and Parkinson’s pathogenesis pathways. It is important to mention here that the *PARK7* gene was identified as differentially expressed in all OS studies and samples we have analysed and the upregulation of P*ARK7* is a constant finding in OS cases. Therefore, activation of this pathway seems to be feasible and functionally plausible. *PARK7* is a gene that supports cell survival, and its upregulation has been shown to be related to aggressive brain cancer progression [21]. The functional deficiency of *PARK7* and mutations in this gene cause autosomal recessive early-onset Parkinson’s disease.

### Gene set enrichment analysis (GSEA) pathway enrichment

**FIGURE 11:**
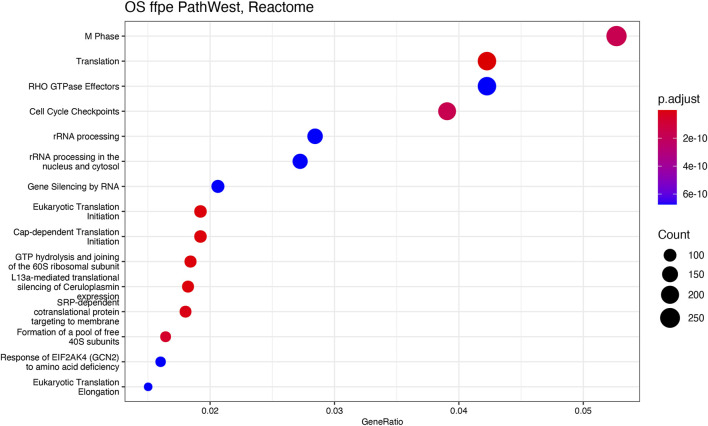
Reactome analysis. The y-axis represents gene ratio values and x-axis represents *p*-value (p adjust). Related pathways with OS are shown on X-axis.

GSEA pathway enrichment was carried out to describe the common themes in the activated gene lists. This is based on the gene set enrichment comparison by using the ranked gene list of the differential expression output. This way only statistically significant differences will be analysed against the existing genes sets [22]. In the OS samples, with the GSEA analysis, it was observed that many gene sets related to the cell cycle and mitosis regulation indicating presence of the malignant disease in OS in comparison to normal. To visualise the GSEA analysis results, we developed dot plots to illustrate the gene number and the statistical significance level for different gene sets and pathway that were activated in OS samples. Figure 11 shows the reactome pathways that are enriched in OS samples. Top 15 highly significant reactome pathways are shown with corresponding adjusted *p*-value and normalized count (Figure 11). Some of the major identified pathways in the plot are protein translation, cell cycle check points, gene silencing and keratinization.

**FIGURE 12:**
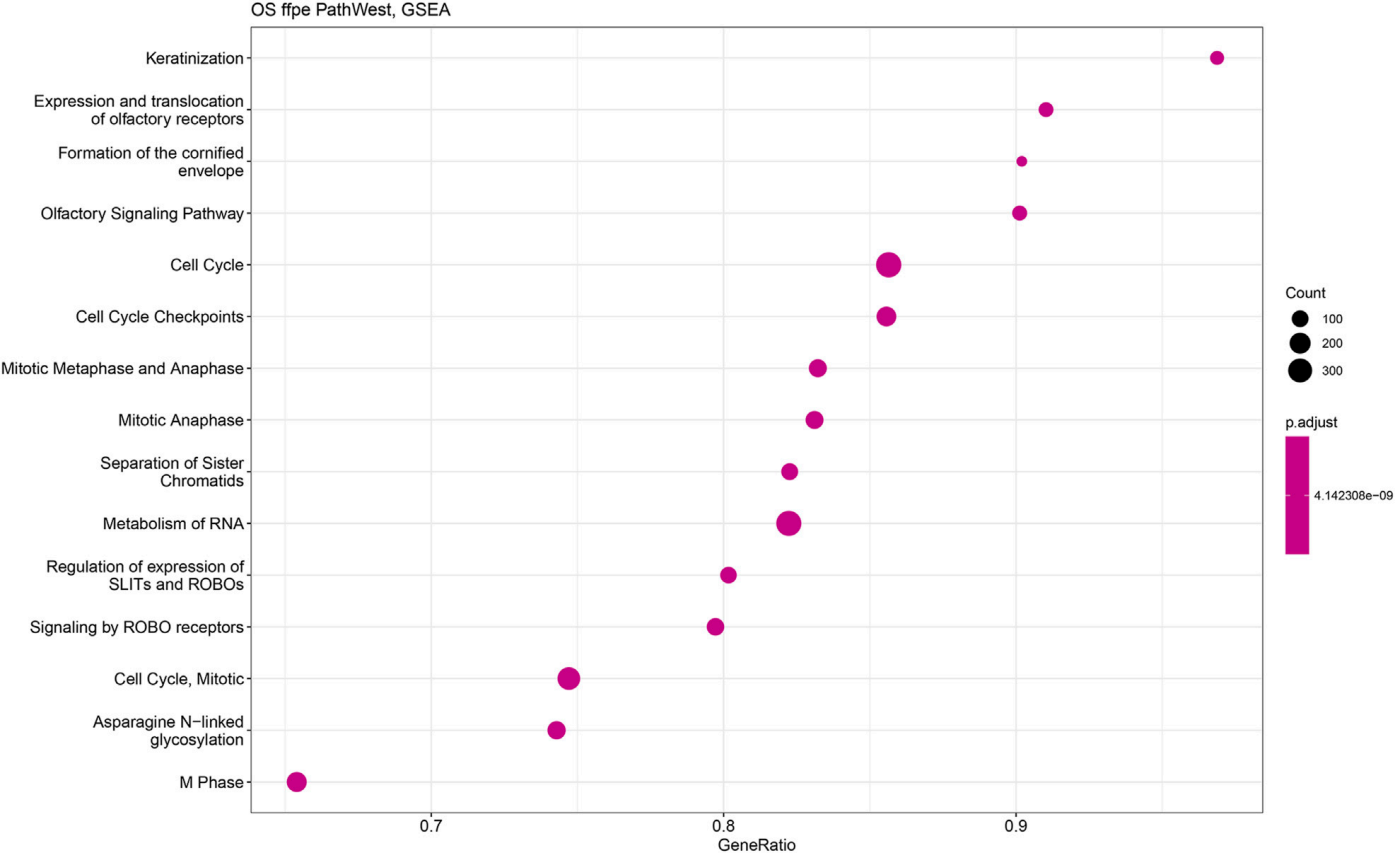
Gene set enrichment analysis of PathWest FFPE samples. The y-axis represents gene ratio values and x-axis represents *p*-value (p adjust). Related pathways with OS are shown on X-axis.

Figures 11, 12 show statistical significance and gene numbers in different GSEA pathways. These illustrations combine the magnitude of the gene activation with the statistical significance. In both the figures we see the activation of cell cycle related pathways and mitosis signals that all reflect the activated malignant processes (Figure 12). Therefore, the genes we identified as differentially expressed in osteosarcoma samples with some indicated in Table 2 is reflective of the malignant process. Functional analysis of the gene activation networks can be even more specific and can go from canonical pathways to the practical analysis of the disease pathologies. Gene set enrichment data were further analysed in the context of known pathologies and diseases by using disease ontology annotation. Interestingly, our dataset only indicated malignant diseases including different cancers, bone development and movement disease (Figure 9). Most remarkably the top disease ontology matches were osteosarcoma and bone cancer.

## Discussion

### Highly upregulated genes in OS

*IFITM5* (Interferon-induced transmembrane protein 5) gene was overexpressed in osteosarcoma samples compared to normal samples. It has been shown that *IFITM5* is expressed predominantly in bone tissue and plays a key role in the formation and mineralisation of bone [23, 24]. In particular, several studies have shown that *IFITM5* is overexpressed in osteosarcoma and also it was reported that the overexpression of IFITM5 has been shown to promote the growth and invasion of osteosarcoma cells *in vitro* and *in vivo* [24, 25]. The exact mechanism by which *IFITM5* promotes the development and progression of osteosarcoma is still not fully understood. However, it is thought that *IFITM5* may regulate the activity of osteoblasts and osteoclasts, which are cells that form and break down bone tissue, respectively. In addition, *IFITM5* may modulate the expression of genes that are important for cell proliferation, invasion, and survival. Overall, while more research is needed to fully understand the role of *IFITM5* in bone cancer, these findings suggest that *IFITM5* may be a potential target for the development of new treatments for osteosarcoma and other bone cancers.

Collagen triple helix repeat containing 1 (*CTHRC1*) is a gene that has been repeatedly shown to be overexpressed in osteosarcoma and is not only involved in carcinogenesis but is also a prognostic marker for malignancy, progression, and OS survival [26]. The high expression of *CTHRC1* has been shown to be correlated with differentiation, recurrency, chemotherapy response, and metastasis in patients with OS [27]. In addition, the survival analysis suggested that high expression of *CTHRC1* in OS patients correlates with a significantly shorter survival time. *CTHRC1* is related to metastasis development and osteosarcoma invasion. Recent research has shown that *CTHRC1* plays an important role in osteosarcoma progression. Lentivirus-mediated short hairpin RNA (shRNA) against *CTHRC1* significantly inhibited cell proliferation and colony formation in U2OS and SW1353 cells [26]. Flow cytometry assay showed that knockdown of *CTHRC1* increased the cell percentage of G0/G1 phase, resulting in cell cycle arrest in U2OS cells. Moreover, *CTHRC1* silencing induced the cell cycle arrest by a decrease in the cell percentage in G0/G1 phase and increased in G2/M phase in SW1353 cells. In addition, crystal violet staining suggested *CTHRC1* silencing inhibited migration of U2OS and SW1353 cells. Downregulation of *CTHRC1* would be an excellent target to stop the metastases of osteosarcoma.

Pannexin 3 is a member of the pannexin family of proteins and observed to be highly expressed in osteosarcoma compared to normal sample. *PANX3* regulates bone growth and differentiation [28] and is expressed in cartilage and regulates chondrocyte proliferation and differentiation [28]. A phenotypic analysis of Panx3-KO mice has indicated that *PANX3* regulates the terminal differentiation of chondrocytes by promoting vascular endothelial growth factor (VEGF) and matrix metalloproteinase (MMP13) [29]. Therefore, *PANX3* is directly involved in the proliferation of the bone progenitor cells. In our studies, we observed *PANX3* was overexpressed in OS samples every time.

The next genes in our list with significant upregulation was the matrix metallopeptidase 13 (*MMP13*). This gene is very well known to be involved in the development of osteosarcoma [30, 31]. *MMP13* is involved in aggressive invasion and migration of the OS. It has been repeatedly shown that inhibition of the MMP13 expression will stop the invasion and growth of the OS [32]. Moreover, MMP13 interacts with other MMPs to form a network for osteosarcoma genes. *MMP13, MMP2, and MMP14* have been identified to interact with each other and to promote the progression and invasion of osteosarcoma [33, 34]. This network also interacts with the COL gene family. Furthermore, the upregulation of matrix metallopeptidase genes, including *MMP1, MMP2, MMP9, MMP11*, and *MMP16*, has been observed in osteosarcoma (OS) [35]. Previous studies have demonstrated the overexpression of these genes not only in OS but also in various types of cancers, where they play a crucial role in cancer survival [36, 37]. Consequently, MMPs could serve as promising diagnostic markers and potential drug targets for osteosarcoma.

*TMEM119* (transmembrane protein 119) was observed to be highly upregulated in osteosarcoma derived samples as compared to healthy samples. The level of *TMEM119* protein expression was shown to be strongly associated with tumour size, clinical stage, distant metastases, and overall survival time [38]. Moreover, the gene set enrichment analysis revealed that *TMEM119* expression in osteosarcoma tissues is positively correlated with cell cycle, apoptosis, metastasis and TGF-β signaling. The reduction of the *TMEM119* expression in U2OS and MG63 cells using small interfering RNA revealed that downregulation of *TMEM119* could inhibit the proliferation of osteosarcoma cells by inducing cell cycle arrest in G0/G1 phase and apoptosis [38]. It was also found that *TMEM119* knockdown significantly inhibited cell migration and invasion and decreased the expression of TGF-β pathway-related factors. Several genes of the TMEM family have been shown to predict the survival of osteosarcoma patients.

Another important gene centromere protein F (*CENPF*) was found to be overexpressed in OS samples in compared to normal samples. *CENPF* has shown to play a key role in regulating the cell cycle and it was also shown that the increased level contributed to accelerate the cell proliferation by mediating apoptosis and cell cycle in OS [39].

### Haemoglobin related genes are downregulated in OS

Similarly, *HBA1, HBA2,* and *HBB* were downregulated in osteosarcoma samples. These are genes that encode haemoglobin subunits and are additionally involved in the malignancies. It was shown that overexpression of *HBA1* and *HBB* inhibits the cell proliferation, induces cellular apoptosis and block the cell cycle at the G2/M phase [40]. *HBB* and *HBA1* are now anti-metastatic factors in other cancers [40, 41] and the downregulation of these genes may be indicative for enhanced formation of metastases. *HBA1* and *HBB* mediate apoptosis and growth arrest on malignant cells, therefore the upregulation of these genes might be beneficial for the OS patients.

### FosB and cell cycle check point protein G0S2 are reduced in OS

*FosB* is a gene named as FBJ murine osteosarcoma viral oncogene homolog B and this gene was downregulated in OS samples. *FosB* is thought to play a role in the development and progression of osteosarcoma by promoting the proliferation and survival of cancer cells. In addition, *FosB* has been found to be involved in the regulation of genes that are important for bone formation, such as osteocalcin and collagen. This suggests that *FosB* may also contribute to the development of osteosarcoma by altering the normal processes of bone formation and remodeling [42, 43]. Overall, while the exact role of *FosB* in osteosarcoma is still being studied, there is evidence to suggest that it may be a potential target for the development of new treatments for this aggressive form of bone cancer. The overexpression of *FosB* gene attenuated lung cancer growth and induced the death of the cancer cells. The downregulation of *FosB* has been shown to be negatively correlated with the cancer grade. Interestingly, the studies from other groups have found that *FosB* is a tumour suppressor gene [44]. Research has shown that the expression of *FosB* is reduced in osteosarcoma cells compared to normal bone cells.

*G0S2* is a G0/G1 switch gene 2 which was observed to be downregulated in osteosarcoma. It is a basic protein that inhibits the proliferation of stem cells. *G0S2* gene is a switch that has been reported to be involved in migration and invasiveness of the malignant cells [45, 46]. *G0S2* gene controls the cell cycle and the down regulation of this gene in the OS samples possibly indicates the mechanisms how OS evades the cell cycle control [47].

This study indicates that even using the partially degraded FFPE samples and with variable quality, the signature of the molecular genetic changes that are indicative for osteosarcoma can be identified. This finding validates our approach and makes the genes listed in Table 2 highly accurate for the OS analysis. The number of similar DEGs that exactly matched across our different independent studies were 530, this number seems to be low in comparison to the observed DEGs in some populations. This could be due to the heterogenic nature of our samples. However, the listed genes do not match with the top DEGs published by Yang et al., in 2014 [48]. It might be due to the heterogeneity of samples and the type of control samples used for the comparison. The enrichment of the GSEA sets and its comparison to the disease ontology showed very clearly that the transcriptional profile we have discovered is related to the osteosarcoma and the genes we have identified are relevant for the OS pathology. GSEA enrichment indicated highly significant enrichment of the genetic networks related to osteosarcoma, bone cancer and connective tissue cancer. The first part of our analysis can be concluded that the FFPE sample analysis resulted in functionally meaningful and feasible results as we were able to identify the gene expression profile that is recognised by the complex computational enrichment analysis as osteosarcoma molecular network. Moreover, genes in Table 2 that is our formal list of statistically significant genes, contains many genes that are identified malignancy genes in literature. These malignancy genes are involved in osteosarcoma, other sarcomas, and in brain cancer.

## Conclusion

Although the prevalence of osteosarcoma is relatively modest compared to other human cancers, the degree of malignancy is extremely high and poses a serious hazard to young patients [2, 3, 6]. The fact that neither the newly developed gene targeting therapy nor immunotherapy, which have been showing inspiring clinical effects in many other tumours, have received a positive response in osteosarcoma, has contributed no notable improvement in patient survival over the past 30 years [6, 49]. It is important to explore the genetics of osteosarcoma in order to screen for prospective genes involved in cancer development and find promising targets. Analysis of differentially expressed gene expression could be a first step to identifying and establishing the marker and may potentially lead to the development of a potential drug target specific to osteosarcoma. From this study we were able to report the top upregulated and downregulated genes specific to OS in comparison to normal tissue which could assist to develop an efficient prognostic marker and therapeutic intervention specific to osteosarcoma.

We observed 530 differentially expressed genes in OS comparison to normal across our four different studies. The top highly expressed genes in OS comparison to normal controls are mostly found to be associated with extra cellular matrix related genes and have shown promise as clinical biomarker and therapeutics target in OS which needs further evaluations in preclinical models. Nevertheless, additional data analysis is required using fresh bone samples and validation of differential gene expression with qPCR and immunocytochemistry. The challenge lies in obtaining fresh bone samples, as not all patients undergo surgery. Another limitation in our study was the heterogeneous nature of the samples in terms of treatment; some underwent drug therapy, and such treatments may impact the gene expression pattern.

## Data Availability

The datasets presented in this study can be found in online repositories. The names of the repository/repositories and accession number(s) can be found below: https://www.ncbi.nlm.nih.gov/, GSE253548.
