# Supplementary material for: The whole transcriptome analysis using FFPE and fresh tissue samples identifies the molecular fingerprint of osteosarcoma
Source: Exp Biol Med (Maywood). 2024 Jun 20;249:10161. doi: 10.3389/ebm.2024.10161 (PMC11222325; doi:10.3389/ebm.2024.10161)
Supplement: Supplementary file 1 [file Table1.DOCX]

**Table S1: Sample detail**

| **Sample_Id** | **Sex** | **SampleGroup** | **Date of birth** | **Age at onset** | **status** | **Age at death** | **Chemotherapy** |
| --- | --- | --- | --- | --- | --- | --- | --- |
| Q01B033022AB2 | F | TUMOUR | 13/04/1985 | 16 | deceased | 17 | chemo |
| Q02B032169YB12 | F | NORMAL | 30/07/1988 | 14 | deceased | 17 | chemo |
| Q02B032169YB18 | F | TUMOUR | 30/07/1988 | 14 | deceased | 17 | chemo |
| Q04B025963TA1 | M | NORMAL | 10/02/1988 | 16 | alive | NA | chemo |
| Q04B025963TB28 | M | TUMOUR | 10/02/1988 | 16 | alive | NA | chemo |
| Q05B005169WA1 | F | NORMAL | 15/11/1995 | 10 | alive | NA | chemosurg |
| Q05B005169WB7 | F | TUMOUR | 15/11/1995 | 10 | alive | NA | chemosurg |
| Q05B009812WA29 | M | TUMOUR | 1/03/1983 | 21 | alive | NA | chemosurg |
| Q05B009812WA32 | M | NORMAL | 1/03/1983 | 21 | alive | NA | chemosurg |
| Q05B030211MA29 | M | TUMOUR | 16/06/1988 | 17 | alive | NA | chemo |
| Q08B047467NA18 | M | TUMOUR | 20/02/1994 | 14 | alive | NA | chemosurg |
| Q08B047467NA3 | M | NORMAL | 20/02/1994 | 14 | alive | NA | chemosurg |
| Q09B042936FB21 | M | TUMOUR | 14/01/1992 | 17 | alive | NA | chemosurg |
| Q09B042936FB9 | M | NORMAL | 14/01/1992 | 17 | alive | NA | chemosurg |
| Q10B040965MA4 | F | TUMOUR | 11/09/1990 | 20 | alive | NA | chemosurg |
| Q10B040965MA55 | F | NORMAL | 11/09/1990 | 20 | alive | NA | chemosurg |
| Q11B045903JA31 | M | NORMAL | 1/02/1985 | 25 | alive | NA | chemosurg |
| Q11B045903JA5 | M | TUMOUR | 1/02/1985 | 25 | alive | NA | chemosurg |
| Q12B019249NA28 | F | TUMOUR | 7/10/1992 | 20 | deceased | 21 | chemosurg |
| Q12B019249NA34 | F | NORMAL | 7/10/1992 | 20 | deceased | 21 | chemosurg |
| Q12B042591TA1 | F | TUMOUR | 20/08/1995 | 16 | deceased | 18 | chemosurg |
| Q12B044305AA16 | M | NORMAL | 13/10/1988 | 26 | alive | NA | chemo |
| Q12B044305AA45 | M | TUMOUR | 13/10/1988 | 26 | alive | NA | chemo |
| Q13B003269YA35 | F | NORMAL | 20/08/1995 | 16 | deceased | 18 | chemosurg |
| Q13B004130DA3 | M | NORMAL | 3/09/1990 | 22 | deceased | 26 | chemosurg |
| Q13B004130DA9 | M | TUMOUR | 3/09/1990 | 22 | deceased | 26 | chemosurg |
| Q13B008611LA13 | M | TUMOUR | 5/03/1997 | 15 | alive | NA | chemosurg |
| Q13B008611LA5 | M | NORMAL | 5/03/1997 | 15 | alive | NA | chemosurg |
| Q13B011918YB10 | M | TUMOUR | 30/12/1993 | 19 | alive | NA | chemosurg |
| Q13B011918YB36 | M | NORMAL | 30/12/1993 | 19 | alive | NA | chemosurg |
| Q13B012216BA21 | M | TUMOUR | 2/11/1993 | 19 | deceased | 24 | chemosurg |
| Q13B012216BA7 | M | NORMAL | 2/11/1993 | 19 | deceased | 24 | chemosurg |
| Q13B020599EB5 | F | TUMOUR | 21/12/1997 | 15 | deceased | 18 | chemosurg |
| Q13B020599EC3 | F | NORMAL | 21/12/1997 | 15 | deceased | 18 | chemosurg |
| Q14B020064NA1 | F | NORMAL | 17/05/1990 | 24 | alive | NA | chemo |
| Q14B020064NA9 | F | TUMOUR | 17/05/1990 | 24 | alive | NA | chemo |
| Q14B024855KA15 | M | TUMOUR | 12/12/1997 | 17 | alive | NA | chemosurg |
| Q14B024855KA29 | M | NORMAL | 12/12/1997 | 17 | alive | NA | chemosurg |
| Q15B001034YA15 | M | TUMOUR | 19/11/1995 | 19 | deceased | 20 | chemo |
| Q15B001034YB1 | M | NORMAL | 19/11/1995 | 19 | deceased | 20 | chemo |
| Q16B027819YA16 | M | NORMAL | 10/12/1997 | 17 | alive | NA | chemosurg |
| Q16B027819YA23 | M | TUMOUR | 10/12/1997 | 17 | alive | NA | chemosurg |
| Q16B037369BA40 | M | NORMAL | 3/08/2003 | 13 | alive | NA | chemosurg |
| Q16B037369BA8 | M | TUMOUR | 3/08/2003 | 13 | alive | NA | chemosurg |
| Q16B040208XA25 | M | NORMAL | 30/03/1999 | 17 | deceased | 18 | chemosurg |
| Q16B040208XA33 | M | TUMOUR | 30/03/1999 | 17 | deceased | 18 | chemosurg |
| Q17B001640BB1 | F | NORMAL | 10/06/1992 | 24 | alive | NA | chemosurg |
| Q17B001640BB5 | F | TUMOUR | 10/06/1992 | 24 | alive | NA | chemosurg |
| Q17B009637RA2 | M | TUMOUR | 27/08/1997 | 19 | alive | NA | chemosurg |
| Q17B009637RB1 | M | NORMAL | 27/08/1997 | 19 | alive | NA | chemosurg |
| Q17B018941HA12 | M | TUMOUR | 3/11/1981 | 36 | deceased | 37 | chemo |
| Q17B018941HB1 | M | NORMAL | 3/11/1981 | 36 | deceased | 37 | chemo |
| Q17B029593MA23 | F | NORMAL | 9/06/1994 | 23 | deceased | 26 | chemo |
| Q17B029593MA7 | F | TUMOUR | 9/06/1994 | 23 | deceased | 26 | chemo |
| Q17B034037YB2 | M | NORMAL | 7/07/1996 | 21 | deceased | 22 | chemo |
| Q17B034037YB20 | M | TUMOUR | 7/07/1996 | 21 | deceased | 22 | chemo |
| Q17B045840YA17 | F | NORMAL | 23/05/2000 | 17 | alive | NA | no |
| Q17B045840YA23 | F | TUMOUR | 23/05/2000 | 17 | alive | NA | no |
| Q17B045995JA29 | F | NORMAL | 30/12/1939 | 78 | deceased | 80 | no |
| Q17B045995JA5 | F | TUMOUR | 30/12/1939 | 78 | deceased | 80 | no |
| Q18B006524DA28 | F | TUMOUR | 29/09/1944 | 74 | deceased | 74 | no |
| Q18B006524DA8 | F | NORMAL | 29/09/1944 | 74 | deceased | 74 | no |
| Q18B009680HA1 | M | TUMOUR | 4/04/2001 | 17 | deceased | 18 | chemo |
| Q18B009680HA12 | M | TUMOUR | 4/04/2001 | 17 | deceased | 18 | chemo |
| Q18B014955AA15 | F | TUMOUR | 30/04/2001 | 17 | deceased | 18 | chemo |
| Q18B014955AA23 | F | NORMAL | 30/04/2001 | 17 | deceased | 18 | chemo |
| Q18B015603EA5 | M | TUMOUR | 29/07/1992 | 26 | alive | NA | chemo |
| Q18B015603EA52 | M | TUMOUR | 29/07/1992 | 26 | alive | NA | chemo |
| Q18B018266YA1 | M | TUMOUR | 5/05/1960 | 58 | deceased | 58 | chemo |
| Q18B018266YE1 | M | NORMAL | 5/05/1960 | 58 | deceased | 58 | chemo |
| Q18B028621HA1 | M | NORMAL | 29/07/1992 | 26 | alive | NA | chemo |
| Q18B028621HA4 | M | TUMOUR | 29/07/1992 | 26 | alive | NA | chemo |
| Q18B034715YA11 | F | NORMAL | 27/12/1999 | 19 | alive | NA | chemo |
| Q18B034715YA6 | F | TUMOUR | 27/12/1999 | 19 | alive | NA | chemo |
| Q18B051017FA1 | M | TUMOUR | 4/11/1949 | 69 | deceased | 71 | chemo |
| Q18B051017FA16 | M | NORMAL | 4/11/1949 | 69 | deceased | 71 | chemo |
| Q19B001229RA22 | F | NORMAL | 1/09/2005 | 13 | alive | NA | chemo |
| Q19B001229RA30 | F | TUMOUR | 1/09/2005 | 13 | alive | NA | chemo |
| Q19B005830YA2 | M | TUMOUR | 19/11/2002 | 17 | alive | NA | chemo |
| Q19B007088FB10 | F | TUMOUR | 11/01/2001 | 17 | alive | NA | chemo |
| Q19B007088FB22 | F | NORMAL | 11/01/2001 | 17 | alive | NA | chemo |
| Q19B013567KA1 | M | TUMOUR | 22/02/1956 | 63 | deceased | 64 | chemo |
| Q19B021879LA19 | M | TUMOUR | 19/11/2002 | 17 | alive | NA | chemo |
| Q19B021879LA21 | M | NORMAL | 19/11/2002 | 17 | alive | NA | chemo |
| Q19B035672TA1 | M | TUMOUR | 5/10/1986 | 33 | alive | NA | chemo |
| Q19B035672TA12 | M | TUMOUR | 5/10/1986 | 33 | alive | NA | chemo |
| Q19B051495PA2 | F | NORMAL | 22/01/2005 | 14 | alive | NA | chemo |
| Q19B051495PB19 | F | TUMOUR | 22/01/2005 | 14 | alive | NA | chemo |
| Q19B052024AB2 | M | TUMOUR | 22/09/1983 | 36 | alive | NA | no |
| Q19B052024AB6 | M | NORMAL | 22/09/1983 | 36 | alive | NA | no |
